# Comparison between vascular age based on brachial-ankle pulse wave velocity or carotid-femoral pulse wave velocity

**DOI:** 10.1038/s41440-025-02281-1

**Published:** 2025-07-18

**Authors:** Tianhui Dong, Fangfang Fan, Hongyu Chen, Zhichen Dong, Leyuan Yang, Yiming Luo, Zifeng Qiu, Ran Fang, Qiwen Zheng, Xiangning Zhang, Jianping Li, Yong Huo, Yan Zhang

**Affiliations:** 1 Department of Cardiology, Peking University First Hospital, Beijing, China; 2 Institute of Cardiovascular Disease, Peking University First Hospital, Beijing, China; 3 Hypertension Precision Diagnosis and Treatment Research Center, Peking University First Hospital, Beijing, China; 4 National Genomics Data Center, China National Center for Bioinformation, Beijing, China; 5 Beijing Institute of Genomics, Chinese Academy of Sciences, Beijing, China; 6 State Key Laboratory of Vascular Homeostasis and Remodeling, Peking University, Beijing, China; 7 NHC Key Laboratory of Cardiovascular Molecular Biology and Regulatory Peptides, Peking University, Beijing, China

**Keywords:** Vascular age, cfPWV, baPWV, Cardiovascular events

## Abstract

Vascular age (VA) as a surrogate of chronological age can improve cardiovascular risk prediction. This study examines which vascular age calculated by brachial-ankle pulse wave velocity (baPWV) or carotid-femoral pulse wave velocity (cfPWV) has a stronger association with the risk of cardiovascular events. This prospective study included 5723 participants from a community-based atherosclerosis cohort in Beijing, China. VA was defined as the predicted age in a multivariable regression model, including classical cardiovascular risk factors, treatment, and pulse wave velocity (baPWV or cfPWV). Residuals by regressing vascular age on chronological age were defined as ∆-age, and the 10th and 90th percentiles of ∆-age were used as cutoffs to define supernormal vascular aging, normal vascular aging, and early vascular aging, respectively. During the median 3.1-year follow-up period, 173 (3.0%) composite endpoints were observed. After adjusting for age and sex, ∆-age calculated by baPWV was significantly associated with cardiovascular risk (hazard ratio [HR]: 1.05, 95% confidence interval [CI]: 1.01–1.09, *p* = 0.025). After adjusting for traditional cardiovascular risk factors, early vascular aging group calculated by baPWV had an increased cardiovascular risk (HR: 1.84, 95% CI: 1.25–2.73, *p* = 0.002), compared with the normal vascular aging group. In contrast, no significant results were observed in the analyses of VA calculated by cfPWV. Since baPWV is a simple and convenient method, VA calculated by baPWV is more valuable for cardiovascular disease risk prediction of large sample population.

**Figure:**
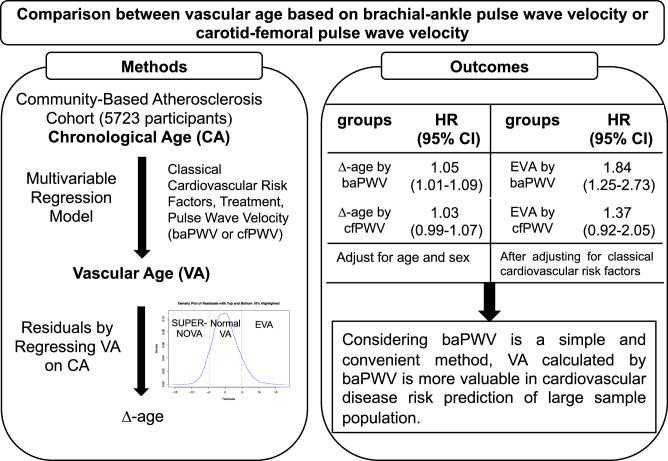
The association between VA based on baPWV or cfPWV and MACE. EVA early vascular aging, normal VA normal vascular aging, SUPERNOVA supernormal vascular aging.

The association between VA based on baPWV or cfPWV and MACE. EVA early vascular aging, normal VA normal vascular aging, SUPERNOVA supernormal vascular aging.

## Abstract (web summary)

The association between VA based on baPWV or cfPWV and MACE. EVA, early vascular aging; normal VA, normal vascular aging; SUPERNOVA, supernormal vascular aging.

## Introduction

Cardiovascular disease (CVD) is a leading cause of morbidity and mortality, making it a major burden of global public health [1]. Various cardiovascular risk prediction models by traditional risk factors have been advocated to identify high-risk populations and guide treatment [2, 3]. Vascular aging plays an important role in the development of CVD, characterized by arteriosclerosis, atherosclerosis, and vascular calcification [4]. Vascular age (VA), better reflecting the health status of vascular trees compared to chronological age, is thought to be an indicator of vascular aging. It has also been investigated and proven that VA can improve cardiovascular risk prediction and be a surrogate of chronological age in CVD risk functions [5–8]. Some scholars have demonstrated that VA based on pulse wave velocity (PWV) can classify the general population into early vascular aging (EVA), normal vascular aging (normal VA), and supernormal vascular aging (SUPERNOVA), which were important concepts for identifying potential high- and low- cardiovascular risk subgroups [9–11].

PWV is recognized and established as an index of arterial stiffness that can independently predict the risk of cardiovascular events. Carotid-femoral pulse wave velocity (cfPWV) has been validated, recommended, and used in Europe, Australia, and the USA for clinical and basic investigators, including VA calculations [12–14]. And another common marker that reflects arterial stiffness is brachial-ankle pulse wave velocity (baPWV), showing prognostic predictability of CVD [15–17]. Studies have promoted that baPWV had a good correlation with cfPWV, and both were significantly associated with cardiovascular risk factors [18, 19].

VA combining classical risk factors with cfPWV and VA combining classical risk factors with baPWV both showed better predictability of cardiovascular events than that only by classical risk factors [9, 11]. Although cfPWV was extensively referenced and proposed for the estimation of arterial stiffness, it requires professional training for operators and exposure to the inguinal region for patients [20]. Instead, baPWV has been proven and used in clinical practice as a simple, automatic, and convenient method [21–23]. The predictive performance of VA by baPWV or cfPWV for the same population is still unknown.

Therefore, we conducted the study to compare the predictability of VA based on baPWV and VA based on cfPWV for cardiovascular risk predictions and determine which has a stronger association with the risk of cardiovascular events in a community-based population in China.

Point of view

- Clinical relevance: Vascular age based on baPWV is more related with CVD risk than vascular age based on cfPWV.
- Future direction: Comparison between vascular age based on baPWV and vascular age based on cfPWV in different population, including the European population needs to be studied.
- Consideration for the Asian population: Considering that the detection of baPWV is simpler and more convenient, we recommend baPWV to evaluate the long-term cardiovascular risk in the Asian population.

## Methods

### Study population

The subjects were recruited from an atherosclerosis cohort conducted in the Pingguoyuan and Gucheng communities in Beijing, China. And the cohort initially investigated 9540 residents ≥40 years old from December 2011 to April 2012, which have been reported in detail previously [24]. We further performed follow-up surveys until the end of 2021. And the follow-up results in 2018 were used as the baseline data in this study. In brief, a total of 6568 subjects were reviewed onsite. Among them, 443 subjects without complete baseline information and 402 subjects with pre-existing myocardial infarction or stroke were excluded. Finally, a total of 5723 subjects were included in the analysis.

This study was approved by the Human Research Ethics Committee of Peking University First Hospital. The protocols were conducted in accordance with institutional guidelines and followed the principles of the Declaration of Helsinki. Written informed consent was obtained from all subjects.

### Data collection

The methods of baseline data collection of this cohort study have been described in detail elsewhere [25]. In detail, standardized questionnaires were adopted for interviewing all subjects’ information about sociodemographic status, education, occupation, diet, lifestyle, health behaviors, and medical history by uniformly trained researchers.

Current smoking was defined as smoking at least one cigarette per day for at least half a year. Body mass index (BMI) was calculated by dividing body weight (in kg) by the square of height (in m). Peripheral blood pressure was measured by well-trained researchers using an Omron HEM-7130 electronic sphygmomanometer following a standard method, and the average value of three measurements was used for analysis.

Blood samples were taken from subjects in a fasting state for at least 12 hours in the morning. The HITACHI 7100 Automatic Analyzer (Hitachi Co., Tokyo, Japan) was used to measure indicators, including fasting blood glucose (FBG), total cholesterol (TC), low-density lipoprotein cholesterol (LDL-C), triglycerides (TG), high-density lipoprotein cholesterol (HDL-C), serum creatinine (Scr). Estimated glomerular filtration rate (eGFR) was calculated by the Chronic Kidney Disease Epidemiology Collaboration (CKD-EPI) equation: Female: Scr ≤0.7 mg/dl, eGFR = 144 x (Scr/0.7)^-0.329^ x (0.993)^Age^; Scr > 0.7 mg/dl, eGFR = 144 x (Scr/0.7)^-1.209^ x (0.993)^Age^; Male: Scr ≤0.9 mg/dl, eGFR = 141 x (Scr/0.9)^-0.411^ x (0.993)^Age^; Scr > 0.9 mg/dl, eGFR = 141 x (Scr/0.9)^-1.209^ x (0.993)^Age^ [26]. All operations were performed in accordance with standardized operating procedures and were carried out by trained and experienced operators.

Hypertension was defined as a systolic blood pressure (SBP) of ≥140 mmHg or a diastolic blood pressure (DBP) of ≥90 mmHg [27], or any self-reported history of hypertension or current treatment with anti-hypertensive medications. Diabetes mellitus was defined as an FBG concentration of ≥7.0 mmol/L, a 2 h oral glucose tolerance test concentration of ≥11.1 mmol/L [28], or any self-reported history of diabetes mellitus or current treatment with anti-diabetes medications. Dyslipidemia was defined as a TG concentration of ≥1.7 mmol/L (150 mg/dL), a TC concentration of ≥5.18 mmol/L (200 mg/dL), an LDL-C concentration of ≥3.37 mmol/L (130 mg/dL), an HDL-C concentration of <1.04 mmol/L (40 mg/dL) [29], or any self-reported history of hyperlipidemia, or current treatment with lipid-lowering medications. CVD was defined as a self-reported history of myocardial infarction or stroke (including transient ischemic attack). A family history of CVD was defined as at least a first-degree relative (including parents, siblings, and children) with CVD.

The 10-year CVD risk was estimated using the China-PAR model. Variables in the China-PAR model include sex, age, geographic region (northern or southern China, as divided by the Yangtze River), urbanization (urban or rural), waist circumference, TC, HDL-C, treated or untreated SBP, diabetes, current smoker, and family history of CVD [3].

### Definition of cardiovascular events

The major adverse cardiovascular event (MACE) was a composite of non-fatal myocardial infarction, non-fatal stroke, and cardiovascular mortality. Data on all participants’ myocardial infarction, stroke, and cardiovascular mortality until Dec 31, 2021 were collected from the Chinese Center for Disease Control and Prevention (National Mortality Surveillance System) and the Beijing Municipal Health Commission (Inpatient Medical Record Home Page System). The International Classification of Diseases in 10th Revision (ICD-10) was used to classify the leading cause of death, myocardial infarction and stroke (Supplementary Table).

### Measurement of baPWV and cfPWV

BaPWV and cfPWV were both measured by trained operators following standardized protocols in all participants after ≥5 min of rest in an examination room with quiet and temperature control.

BaPWV was measured using an automatic waveform analyzer (BP-203RPE III, Colin-Omron, Co., Ltd., Tokyo, Japan) [30]. The subjects were in the supine position, and four cuffs were wrapped around the bilateral brachia and ankles and then connected to a plethysmographic sensor and oscillometric pressure sensor. The pulse waveforms were then recorded and the baPWV value was automatically generated. The higher baPWV of both the left and right sides was used for subsequent analyses after examination.

CfPWV was measured using an automated system (Pulse Pen, DiaTecne, Milan, Italy) [31]. The right carotid and femoral waveforms were acquired simultaneously with two pressure-sensitive transducers, and the transit time of the pulse was calculated using the system software. The distances of the carotid and femoral artery, carotid and sternal angle, and sternal angle and femoral artery were measured. The cfPWV value was computed using the formula [PWV = distance (m)/time (s)], which was at least twice measured. If the difference between the two results exceeded 0.5 m/s, a third measurement was taken. The average value was then applied.

### Definition of vascular age, EVA and SUPERNOVA

VA was defined as the predicted age in a multivariable regression model that included classical cardiovascular risk factors, treatment, and PWV (baPWV or cfPWV) as independent variables [9]. A backward stepwise approach made the variable selection with multicollinearity checked by variable inflation factors (VIF, variables excluded when VIF > 10) which was performed in VA model based on baPWV and VA model based on cfPWV respectively. Variables showing a nonlinear relationship with age were transformed by smoothing splines using generalized additive models (knots chosen by 10-fold cross-validation). The final variables for calculating VA based on baPWV include sex, SBP, DBP, FBG, TG, LDL-C, waist circumference, BMI, current smoking, treatment of hypertension, and baPWV. And the final variables for calculating VA based on cfPWV include sex, SBP, DBP, FBG, TG, LDL-C, waist circumference, BMI, heart rate, current smoking, treatment of hypertension and dyslipidemia, and cfPWV.

∆-age was estimated as the residuals by regressing VA on chronological age [32, 33]. The 10th and 90th percentiles of ∆-age were used as cutoffs to define SUPERNOVA, normal VA, and EVA groups, respectively.

### Statistical analyses

The baseline characteristics of subjects were expressed as mean ± SD or median with interquartile range and compared among three groups (SUPERNOVA, normal VA, and EVA) using the *t* test or Kruskal‐Wallis rank test for continuous variables as appropriate. For categorical variables, data were expressed as frequency (percentage) and compared among three groups (SUPERNOVA, normal VA, and EVA) using the chi‐square test or Fisher exact test as appropriate. The correlation and agreement between VA by baPWV and cfPWV were assessed using the Pearson correlation coefficient and Bland–Altman plot.

The association between ∆-age as a continuous variable or three groups (SUPERNOVA, normal VA, and EVA) and outcomes were assessed by Cox proportional hazards regression. Covariates in Model 1 included age and sex; those in model 2 included traditional cardiovascular risk factors, including age, sex, current smoking, BMI, hypertension, diabetes, dyslipidemia, anti-hypertensive treatment, lipid-lowering treatment, hypoglycemic treatment, family history of CVD, and eGFR.

A two-tailed p-value of <0.05 was considered statistically significant in all analyses. R software (version 4.3.2, http://www.R-project.org/) was used to perform the statistical analyses.

## Results

### Baseline clinical characteristics

A total of 5723 subjects finally participated in the study during the median 3.1-year follow-up period. There were 173 (3.0%) composite endpoints observed, of which 35 (0.6%) were acute myocardial infarction, 21 (0.4%) cardiovascular mortality, and 130 (2.3%) stroke. The clinical characteristics of the patients in the three groups (SUPERNOVA, normal VA, and EVA) were shown in Table 1.

**Table 1:** Baseline Clinical Characteristics of Participants all and in three groups by baPWV/cfPWV-based VA

Variables	Overall population (5723)	SUPERNOVA (573)	Normal VA (4577)	EVA (573)	*P*-value
**Chronological age, y**	61(57–66)				
baPWV-based VA		63(58–67)	61(57–66)	63(58–67)	<0.001
cfPWV-based VA		63(59–68)	61(57–65)	63(58–68)	<0.001
**Vascular age, y**					
baPWV-based VA	61.7(58.6–65.2)	56.0(54.3–57.9)	61.6(59.0–64.5)	69.0(66.9–71.4)	<0.001
cfPWV-based VA	61.5(58.7–64.8)	56.4(55.0–58.3)	61.4(59.1–64.1)	69.2(67.0–71.8)	<0.001
**∆-age, y**					
baPWV-based VA	−0.10(−2.58 to 2.43)	−5.94(−6.94 to −5.12)	−0.10(−2.02 to 1.86)	6.38(5.44–7.56)	<0.001
cfPWV-based VA	−0.19(−2.58 to 2.33)	−5.67(−6.64 to −5.09)	−0.19(−2.06 to 1.74)	6.48(5.56–7.71)	<0.001
**Male, n (%)**	1881(32.9)				
baPWV-based VA		115(20.1)	1486(32.5)	280(48.9)	<0.001
cfPWV-based VA		124(21.6)	1475(32.2)	282(49.2)	<0.001
**BMI, kg/m**^**2**^	25.0(23.0–27.2)				
baPWV-based VA		24.8(22.9–27.2)	25.0(23.0–27.2)	25.2(23.3–27.3)	0.260
cfPWV-based VA		24.9(22.9–27.4)	25.0(23.0–27.1)	25.3(23.4-27.6)	0.032
**WC, cm**	87(80–93)				
baPWV-based VA		83(77–90)	87(80–93)	90(85–96)	<0.001
cfPWV-based VA		83(77–90)	87(80–93)	90(85–97)	<0.001
**Current smoking, n (%)**	802(14.0)				
baPWV-based VA		99(17.3)	641(14.0)	62(10.8)	0.007
cfPWV-based VA		105(18.3)	627(13.7)	70(12.2)	0.006
**SBP, mmHg**	132.0(121.0–143.3)				
baPWV-based VA		122.7(113.3–134.0)	131.7(121.3–142.3)	143.7(134.0–154.7)	<0.001
cfPWV-based VA		123.7(112.7–134.3)	131.7(121.0–142.0)	145.3(134.3–156.0)	<0.001
**DBP, mmHg**	78.7(72.7–85.3)				
baPWV-based VA		81.0(75.3–88.0)	78.7(72.7–85.3)	75.7(69.0–82.7)	<0.001
cfPWV-based VA		81.3(75.0–88.3)	78.7(73.0–85.3)	75.0(68.7–82.0)	<0.001
**Heart rate, bpm**	76.7(69.7–85.0)				
baPWV-based VA		78.0(71.0–85.7)	76.3(69.3–84.7)	77.3(69.3–87.0)	0.005
cfPWV-based VA		77.0(70.0–84.7)	76.7(69.7–85.0)	76.7(68.3–86.0)	0.519
**TC, mmol/L**	5.35(4.71–6.03)				
baPWV-based VA		5.55(5.05–6.23)	5.36(4.73–6.04)	5.01(4.31–5.71)	<0.001
cfPWV-based VA		5.62(5.08–6.28)	5.35(4.72–6.03)	5.03(4.36–5.72)	<0.001
**TG, mmol/L**	1.38(0.99–1.96)				
baPWV-based VA		1.47(1.02–2.14)	1.38(0.99–1.94)	1.31(0.96–1.96)	0.007
cfPWV-based VA		1.50(1.06–2.21)	1.37(0.98–1.94)	1.31(0.97–1.96)	<0.001
**HDL-C, mmol/L**	1.45(1.25–1.70)				
baPWV-based VA		1.48(1.27–1.72)	1.45(1.26–1.70)	1.41(1.20–1.64)	0.001
cfPWV-based VA		1.48(1.26–1.71)	1.45(1.25–1.70)	1.42(1.21–1.63)	0.002
**LDL-C, mmol/L**	3.43(2.80–4.08)				
baPWV-based VA		3.62(3.06–4.20)	3.44(2.81–4.09)	3.09(2.40–3.84)	<0.001
cfPWV-based VA		3.61(3.06–4.25)	3.44(2.81–4.08)	3.12(2.45–3.85)	<0.001
**FBG, mmol/L**	5.60(5.15–6.30)				
baPWV-based VA		5.30(5.00–5.90)	5.60(5.20–6.30)	6.00(5.40–7.20)	<0.001
cfPWV-based VA		5.30(5.00–5.90)	5.60(5.20–6.30)	5.90(5.30–7.00)	<0.001
**eGFR, mL/min/1.73** ^**m2**^	95.66(88.60–100.80)				
baPWV-based VA		94.21(85.50–99.54)	95.85(89.19–100.96)	95.13(86.75–100.92)	<0.001
cfPWV-based VA		93.53(85.90–98.69)	96.04(89.36–101.05)	94.13(85.12–99.78)	<0.001
**Hypertension, n (%)**	3039(53.1)				
baPWV-based VA		211(36.8)	2365(51.7)	463(80.8)	<0.001
cfPWV-based VA		220(38.4)	2348(51.3)	471(82.2)	<0.001
**Diabetes, n (%)**	1573(27.5)				
baPWV-based VA		115(20.1)	1192(26.0)	266(46.4)	<0.001
cfPWV-based VA		108(18.8)	1210(26.4)	255(44.5)	<0.001
**Dyslipidemia, n (%)**	4644(81.1)				
baPWV-based VA		492(85.9)	3704(80.9)	448(78.2)	0.002
cfPWV-based VA		493(86.0)	3697(80.8)	454(79.2)	0.004
**Anti-hypertensive medications, n (%)**	1934(33.8)				
baPWV-based VA		117(20.4)	1494(32.6)	323(56.4)	<0.001
cfPWV-based VA		116(20.2)	1492(32.6)	326(56.9)	<0.001
**Anti-diabetes medications, n (%)**	850(14.9)				
baPWV-based VA		53(9.2)	637(13.9)	160(27.9)	<0.001
cfPWV-based VA		50(8.7)	647(14.1)	153(26.7)	<0.001
**Lipid-lowering medications, n (%)**	1045(18.3)				
baPWV-based VA		75(13.1)	821(17.9)	149(26.0)	<0.001
cfPWV-based VA		73(12.7)	810(17.7)	162(28.3)	<0.001
**CVD risk, %**	7.32(4.04–12.28)				
baPWV-based VA		5.37(3.35–9.78)	6.98(3.97–11.87)	11.11(7.40–17.09)	<0.001
cfPWV-based VA		5.83(3.48–10.26)	6.95(3.91–11.71)	12.17(7.58–17.39)	<0.001
**baPWV, m/s**	16.16(14.35–18.50)	13.86(12.78–15.12)	16.13(14.49–18.13)	20.18(18.11–22.88)	<0.001
**cfPWV, m/s**	8.15(7.30–9.35)	7.21(6.55–7.92)	8.11(7.32–9.15)	10.53(9.27–12.14)	<0.001

Data were presented as median (IQR) or n (%) of the group. ∆-age indicates the residuals by regressing vascular age on chronological age, *BMI* body mass index, *WC* waist circumference, *SBP* systolic blood pressure, *DBP* diastolic blood pressure, *TC* total cholesterol, *TG* triglycerides, *HDL-C* high-density lipoprotein cholesterol, *LDL-C* low-density lipoprotein cholesterol *FBG* fasting blood glucose, *eGFR* estimated glomerular filtration rate, *CVD* cardiovascular disease, *baPWV* brachial-ankle pulse wave velocity, *cfPWV* carotid-femoral pulse wave velocity, *SUPERNOVA* supernormal vascular aging, *normal VA* normal vascular aging, *EVA* early vascular aging

The ranges of VA based on baPWV/cfPWV were 45.5 to 82.5 years and 45.1 to 81.8 years, respectively. According to the 10th and 90th percentile of ∆-age, VA categorized by baPWV was defined as SUPERNOVA (∆-age < –4.62 years), normal VA (–4.62 years ≤ ∆-age ≤ 4.85 years), and EVA (∆-age > 4.85 years); VA categorized by cfPWV was defined as SUPERNOVA (∆-age < –4.59 years), normal VA (–4.59 years ≤ ∆-age ≤ 4.85 years), and EVA (∆-age > 4.85 years).

As shown in Table 1, EVA group based on baPWV and that based on cfPWV both showed older VA, a higher proportion of male sex, higher SBP and FBG levels, larger waist circumference, higher cfPWV value and baPWV value, and higher CVD risk. SUPERNOVA groups both had higher DBP levels, a higher percentage of dyslipidemia, and current smoking.

### Correlation and agreement comparison between VA based on baPWV and cfPWV

**Fig. 1:**
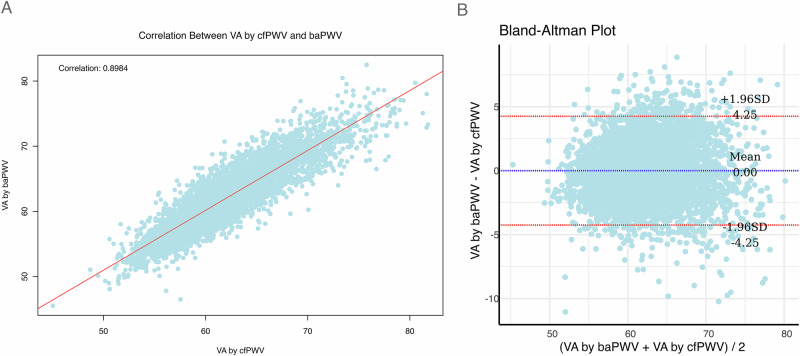
**Correlation and agreement comparison between VA based on baPWV and cfPWV**. Good Correlation (**A**) and agreement (**B**) between VA based on baPWV and VA based on cfPWV were observed. VA vascular age, baPWV brachial-ankle pulse wave velocity; cfPWV carotid-femoral pulse wave velocity

Good correlation and agreement between VA based on baPWV and VA based on cfPWV were found (correlation: *r* = 0.8984; Fig. 1A; agreement: mean difference: 0.00 ± 4.25, 95% confidence interval (CI): –0.056 ~ 0.056, *p* > 0.05; Fig. 1B). More than 95% of points fall within the upper and lower 95% limits of agreement.

### Comparison of association between VA based on baPWV/cfPWV and cardiovascular events

∆-age based on baPWV as a continuous variable was significantly and increasingly associated with the risk of MACE after adjusting for age and sex, and every 1-year increase associated with a rise of 5% in MACE risk (hazard ratio [HR]: 1.05, 95% CI: 1.01–1.09, *p* = 0.025; Table 2). After adjusting for traditional cardiovascular risk factors, ∆-age based on baPWV was still significantly associated with the risk of MACE (HR: 1.04, 95% CI: 1.00–1.09, *p* = 0.044; Table 2). ∆-age based on cfPWV as a continuous variable showed every 1-year increase led to a rise of 3%, which is not statistically significant (HR: 1.03, 95% CI: 0.99–1.07, *p* = 0.097; Table 3).

**Table 2:** Cox proportional hazards regression analyses for the association between baPWV-based VA and the risk of cardiovascular events

Outcomes	VA Categories	Event *n* (%)	Model 1		Model 2		
			HR (95%CI)	*P*-value	HR (95%CI)	*P*-value	
MACE	∆-age	173(3)	1.05(1.01–1.09)	0.025	1.04(1.00–1.09)		0.044
	SUPERNOVA	14(2.4)	0.90(0.52–1.56)	0.704	0.84(0.48–1.47)		0.536
	Normal VA	123(2.7)	reference	-	reference		-
	EVA	36(6.3)	1.95(1.34–2.84)	<0.001	1.84(1.25–2.73)		0.002
CVD mortality	∆-age	21(0.4)	1.10(0.98–1.22)	0.104	1.08(0.96–1.21)		0.193
	SUPERNOVA	0(0.0)	-	-	-		-
	Normal VA	16(0.3)	reference	-	reference		-
	EVA	5(0.9)	1.87(0.68–5.15)	0.226	1.64(0.56–4.77)		0.364
AMI	∆-age	35(0.6)	1.01(0.93–1.10)	0.820	1.01(0.92–1.11)		0.806
	SUPERNOVA	2(0.3)	0.60(0.14–2.53)	0.484	0.54(0.12–2.31)		0.404
	Normal VA	27(0.6)	reference	-	reference		-
	EVA	6(1.0)	1.50(0.61–3.66)	0.374	1.44(0.57–3.64)		0.443
Stroke	∆-age	130(2.3)	1.05(1.00–1.10)	0.034	1.05(1.00–1.10)		0.044
	SUPERNOVA	12(2.1)	1.07(0.59–1.96)	0.822	0.99(0.54–1.85)		0.987
	Normal VA	89(1.9)	reference	-	reference		-
	EVA	29(5.1)	2.14(1.40–3.27)	<0.001	2.08(1.33–3.24)		0.001

Model 1 was adjusted for age and sex; model 2 was adjusted for age, sex, current smoking, body mass index, hypertension, diabetes, dyslipidemia, anti-hypertensive treatment, lipid-lowering treatment, hypoglycemic treatment, family history of cardiovascular disease, and estimated glomerular filtration rate

*baPWV* brachial-ankle pulse wave velocity, *VA* vascular age, *HR* hazard ratio, *MACE* a composite of acute myocardial infarction, stroke, and cardiovascular mortality, *CVD* cardiovascular disease, *AMI* acute myocardial infacrtion, ∆-age indicates the residuals by regressing vascular age on chronological age, *SUPERNOVA* supernormal vascular aging, *normal VA* normal vascular aging, *EVA* early vascular aging

**Table 3:** Cox proportional hazards regression analyses for the association between cfPWV-based VA and the risk of cardiovascular events

Outcomes	VA Categories	Event n (%)	Model 1		Model 2	
			HR (95%CI)	P value	HR (95%CI)	P value
MACE	∆-age	173(3)	1.03(0.99–1.07)	0.097	1.02(0.98–1.06)	0.320
	SUPERNOVA	13(2.3)	0.77(0.43–1.37)	0.373	0.73(0.41–1.31)	0.295
	Normal VA	127(2.8)	reference	-	reference	-
	EVA	33(5.8)	1.56(1.06–2.30)	0.024	1.37(0.92–2.05)	0.122
CVD mortality	∆-age	21(0.4)	1.03(0.93–1.14)	0.564	0.99(0.89–1.11)	0.925
	SUPERNOVA	1(0.2)	0.47(0.06–3.56)	0.464	0.48(0.06–3.79)	0.488
	Normal VA	16(0.3)	reference	-	reference	-
	EVA	4(0.7)	1.28(0.42–3.87)	0.663	0.91(0.29–2.84)	0.872
AMI	∆-age	35(0.6)	1.01(0.93–1.10)	0.751	1.01(0.92–1.10)	0.902
	SUPERNOVA	4(0.7)	1.26(0.43–3.64)	0.673	1.14(0.38–3.36)	0.818
	Normal VA	25(0.5)	reference	-	reference	-
	EVA	6(1.0)	1.51(0.61–3.73)	0.369	1.33(0.52–3.37)	0.554
Stroke	∆-age	130(2.3)	1.04(0.99–1.08)	0.099	1.03(0.98–1.07)	0.262
	SUPERNOVA	9(1.6)	0.72(0.36–1.43)	0.344	0.67(0.33–1.35)	0.262
	Normal VA	95(2.1)	reference	-	reference	-
	EVA	26(4.5)	1.62(1.05–2.52)	0.031	1.44(0.92–2.27)	0.114

Model 1 was adjusted for age and sex; model 2 was adjusted for age, sex, current smoking, body mass index, hypertension, diabetes, dyslipidemia, anti-hypertensive treatment, lipid-lowering treatment, hypoglycemic treatment, family history of cardiovascular disease, and estimated glomerular filtration rate

*cfPWV* carotid-femoral pulse wave velocity, *VA* vascular age, *HR* hazard ratio, *MACE* a composite of acute myocardial infarction, stroke, and cardiovascular death, *CVD* cardiovascular disease, *AMI* acute myocardial infacrtion, ∆-age indicates the residuals by regressing vascular age on chronological age, *SUPERNOVA* supernormal vascular aging, *normal VA* normal vascular aging, *EVA* early vascular aging

When ∆-age was categorized into three VA categories, the subjects in EVA groups by baPWV and cfPWV both experienced a higher risk of MACE compared with normal VA groups after adjusting for age and sex (VA based on baPWV: HR: 1.95, 95%CI: 1.34–2.84, p < 0.001; VA based on cfPWV: HR: 1.56, 95%CI: 1.06–2.30, p = 0.024). The SUPERNOVA groups by baPWV and cfPWV associated with the 10% and 23% risk reduction of MACE, respectively. After adjusting for traditional cardiovascular risk factors, the EVA group based on baPWV also had a statistically increased risk of MACE (HR: 1.84, 95% CI: 1.25–2.73, p = 0.002) compared with the normal VA group. However, no statistically significant results were observed for VA based on cfPWV (HR: 1.37, 95% CI: 0.92–2.05, p = 0.122).

In secondary endpoint analysis, VA categories based on baPWV showed trends with risk differences in cardiovascular mortality, acute myocardial infarction and stroke. However, similar trends with risk differences were only found in stroke endpoint for VA categories based on cfPWV (Table 2, Table 3).

## Discussion

In the present study, we found good correlation and agreement between VA based on baPWV and VA based on cfPWV. When associated with the cardiovascular events, ∆-age by baPWV was positively related to the risk of MACE, that is the risk increased by 5% for a year increase of ∆-age, and after adjusting traditional cardiovascular risk factors, EVA group by baPWV had a 84% increase of MACE risk compared with the normal VA group. However, these results were not found in VA model based on cfPWV, which suggested that VA based on baPWV has a better predictive ability for cardiovascular events than that estimated by cfPWV in the Chinese population.

Previous studies have been investigated that VA can be a surrogate of chronological age in CVD risk functions [5–8]. As the baseline results in this study showed, chronological age among VA categories based on baPWV or cfPWV were similar, and it is difficult to distinguish the degree of vascular aging only according to their chronological age. Vascular age serves as a new indicator that explains to the patients from a perspective of vascular health, which is beneficial for understanding [34]. Several methods for VA calculation have been suggested such as risk-based VA and value-based VA, which were based on cardiovascular risk factors or arterial injury indicators respectively. And arterial injury indicators including carotid intima-media thickness (CIMT), coronary arterial calcification (CAC), PWV, and so on [6].

Subsequently, scholars proposed integrating above methods for VA calculation and studies that combined cardiovascular risk factors and PWV for the calculation of VA showed a higher risk of cardiovascular events in the EVA groups and a lower risk of cardiovascular events in the SUPERNOVA group whether PWV was cfPWV or baPWV [9–11]. However, few studies compared the predictive value of VA based on cfPWV and baPWV simultaneously. This study is the first comparison between VA based on baPWV and VA based on cfPWV. And the results showed that VA based on baPWV had a good correlation and agreement with VA based on cfPWV, which corresponded to previous studies showing a good correlation between baPWV and cfPWV [18, 19], as baPWV and cfPWV are both established indices of arterial stiffness. Then, we found that EVA based on baPWV has a stronger association with the risk of cardiovascular events than EVA based on cfPWV, including the endpoint of MACE and stroke.

CfPWV is mainly used to assess the stiffness of large central arteries such as the aorta, and baPWV reflects the stiffness of both central and peripheral muscular arteries [35, 36]. Abnormality of peripheral vascular diseases reflected by baPWV may indicate an extensive and severe degree of systemic atherosclerosis and, hence, is more related to poorer cardiovascular outcomes shown in this study [37]. Although cfPWV is currently the most validated marker of vascular aging which is accurate and recommended to use [14], it may not be ideal for routine use in clinics because the difficult operations to clinical staff. Additionally, some subjects may feel uncomfortable exposing the inguinal area during the measure process of cfPWV. Considering that the detection of baPWV is simpler and more convenient, it is an advantageous tool for identifying high-risk people in large populations.

Besides, the risk of ASCVD in EVA groups in previous articles were the lowest at baseline results [9, 11], while EVA groups by cfPWV and baPWV in the present study both showed higher CVD risk than the other two groups. The latter is reasonable because vascular aging related to traditional cardiovascular risk factors is the most common and officially recognized mechanism in the progression of CVD [38, 39]. Similarly, study using only baPWV for EVA definition also had a higher proportion of classical cardiovascular risk factors [40]. According to the current classification criteria, the SUPERNOVA group includes individuals with younger VA who still have healthy arteries, whether or not they are exposed to cardiovascular risk factors. Therefore, the SUPERNOVA group may not necessarily have the highest CVD risk. Higher ASCVD risk in the SUPERNOVA group in previous articles may be due to the older chronological age. In fact, the identification of the SUPERNOVA population still needs to be determined by exploring protective molecular pathways or novel biomarkers [41].

Some limitations to our study should be noted. First, the follow-up time of this study is relatively short, with a median 3.1-year follow-up period. The cohort is being followed up continually and the results will be updated. Second, vascular aging is mainly attributed to atherosclerosis, arteriosclerosis, and vascular calcification, which are different pathogenic processes estimated by various indicators [4, 42]. Further studies of VA calculated by different vascular injury indicators, such as CIMT and CAC, combined with traditional cardiovascular risk factors can be carried out to optimize the prediction performance.

### Asian perspectives

Previous studies showed the results that vascular age based on cfPWV in European population and vascular age based on baPWV in Asian population both have higher risk of cardiovascular events in the EVA groups and lower risk of cardiovascular events in the SUPERNOVA group [9–11]. This study have compared the predictive ability of VA based on baPWV and based on cfPWV in Asian population simultaneously and found better performance in VA based on baPWV. This result may indicate the different applicability of PWV in European and Asian populations.

## Conclusion

We found that VA based on baPWV has a better predictive ability for cardiovascular events than that based on cfPWV in this community-based cohort in China. Since baPWV is a simple and convenient method, VA based on baPWV is more valuable for CVD risk prediction of large sample population.

## Supplementary information

Supplementary Table
