# Supplementary material for: Comparison between vascular age based on brachial-ankle pulse wave velocity or carotid-femoral pulse wave velocity
Source: Hypertens Res. 2025 Jul 18;48(9):2315–25. doi: 10.1038/s41440-025-02281-1 (PMC12411221; doi:10.1038/s41440-025-02281-1)
Supplement: Supplementary file 1 — Supplementary Table [file 41440_2025_2281_MOESM1_ESM.docx]

**Supplementary Table The source and ICD-10 code of the cardiovascular events in this study**

| Cardiovascular events | Source | ICD-10 code |
| --- | --- | --- |
| Cardiovascular death | The Chinese Center for Disease Control and Prevention-National Mortality Surveillance System | I00-I99 |
| Myocardial infarction | The Beijing Municipal Health Commission-Beijing inpatient medical record home page systerm | Acute ST-segment elevation myocardial infarction (I21.001-006, I21.101-105, I21.201-211, I21.213-230, I21.301, I21.304, I22.001-003, I22.101-103, I22.801-818), Acute non-ST-segment elevation myocardial infarction (I21.401-404), Other myocardial infarction (I21.302, I21.303, I21.305-308, I21.901, I21.902, I21.907, I21.910, I21.911, I22.901) |
|  | The Chinese Center for Disease Control and Prevention-National Mortality Surveillance System | I21.0, I21.1, I21.2, I21.3, I21.4, I21.9, I22.0, I22.1, I22.8, I22.9 |
| Stroke | The Beijing Municipal Health Commission-Beijing inpatient medical record home page system | Ischemic stroke (I63), Hemorrhagic stroke (I60-I61), Other stroke (I64) |
|  | The Chinese Center for Disease Control and Prevention-National Mortality Surveillance System | I60, I61, I63, I64 |

ICD-10: The International Classification of Diseases in 10th Revision.
